# Motor imagery electroencephalogram classification algorithm based on joint features in the spatial and frequency domains and instance transfer

**DOI:** 10.3389/fnhum.2023.1175399

**Published:** 2023-05-05

**Authors:** Ximiao Wang, Xisheng Dai, Yu Liu, Xiangmeng Chen, Qinghui Hu, Rongliang Hu, Mingxin Li

**Affiliations:** ^1^Institute of Intelligent Systems and Control, Guangxi University of Science and Technology, Liuzhou, China; ^2^School of Electronic Information and Automation, Guilin University of Aerospace Technology, Guilin, China; ^3^Department of Radiology, Jiangmen Central Hospital, Jiangmen, Guangdong, China; ^4^School of Computer Science and Engineering, Guilin University of Aerospace Technology, Guilin, China; ^5^Department of Rehabilitation Medicine, Jiangmen Central Hospital, Jiangmen, Guangdong, China

**Keywords:** brain-computer interface (BCI), motor imagery (MI), joint feature, instance transfer, ensemble learning, kernel mean matching (KMM), transfer learning adaptive boosting (TrAdaBoost)

## Abstract

**Introduction:**

Motor imagery electroencephalography (MI-EEG) has significant application value in the field of rehabilitation, and is a research hotspot in the brain-computer interface (BCI) field. Due to the small training sample size of MI-EEG of a single subject and the large individual differences among different subjects, existing classification models have low accuracy and poor generalization ability in MI classification tasks.

**Methods:**

To solve this problem, this paper proposes a electroencephalography (EEG) joint feature classification algorithm based on instance transfer and ensemble learning. Firstly, the source domain and target domain data are preprocessed, and then common space mode (CSP) and power spectral density (PSD) are used to extract spatial and frequency domain features respectively, which are combined into EEG joint features. Finally, an ensemble learning algorithm based on kernel mean matching (KMM) and transfer learning adaptive boosting (TrAdaBoost) is used to classify MI-EEG.

**Results:**

To validate the effectiveness of the algorithm, this paper compared and analyzed different algorithms on the BCI Competition IV Dataset 2a, and further verified the stability and effectiveness of the algorithm on the BCI Competition IV Dataset 2b. The experimental results show that the algorithm has an average accuracy of 91.5% and 83.7% on Dataset 2a and Dataset 2b, respectively, which is significantly better than other algorithms.

**Discussion:**

The statement explains that the algorithm fully exploits EEG signals and enriches EEG features, improves the recognition of the MI signals, and provides a new approach to solving the above problem.

## 1. Introduction

In recent years, brain-computer interface (BCI) systems have attracted great attention because they can provide another communication channel for people who have lost independent motor ability by decoding neural signals (Wolpaw et al., 2002). This system converts electroencephalography (EEG) collected from the scalp into control commands for computers or other external devices, thereby achieving direct interaction between the brain and external devices (Xu et al., 2018). In EEG-based BCI experiments, motor imagery (MI) is a commonly used task paradigm.

Brain-computer interface based on MI means that there is no actual physical behavior involved, but rather the thoughts in the brain are used to imagine bodily movements, which are then translated into actual operations through a controller. MI activates brain regions that are similar to those activated during actual physical movement (Holmes and Calmels, 2008; Miltona et al., 2008), which can promote the repair or reconstruction of damaged motor pathways, has great application value in the field of rehabilitation. However, due to the small amount of Mi-EEG sample data of a single subject and the large individual differences among different subjects (Manali et al., 2020), the classification model has low accuracy and poor generalization ability in the MI classification task.

To solve these problems, researchers have conducted extensive studies. Due to the asymmetric energy differences in the human spatial brain area during motor imagery, researchers usually use the Common Spatial Pattern (CSP) algorithm to extract distinguishable spatial features from multi-channel EEG signals (Ramoser et al., 2000; Amirhossein et al., 2016). In addition, EEG signals are mainly rhythmic signals, and Event-Related Desynchronization (ERD) (Zhang et al., 2021) and Event-Related Synchronization (ERS) (Bastien et al., 2020) occur within specific frequency bands. At the same time, the CSP algorithm directly filters spatially in the frequency domain, so frequency domain information cannot be ignored for MI classification. Therefore, many scholars have proposed combining spatial and frequency domain features to fully explore EEG and enrich EEG features. For example, Ai et al. (2019) used the CSP algorithm and the local characteristic-scale decomposition (LCD) algorithm to extract spatial and frequency domain features, respectively, and combined the two types of features. Finally, they used the Spectral Regression Discriminant Analysis (SRDA) classifier for classification, achieving an average classification accuracy of 74.5%. However, this method ignores the personalized differences between subjects, resulting in poor generalization ability of the classification model.

In recent years, studies have shown that transfer learning can improve model performance in target tasks by learning and transferring information from source tasks (Feng et al., 2022). Therefore, some researchers hope to reduce the individual differences among different subjects through transfer learning (Cao et al., 2021; Feng et al., 2023), so as to improve the performance of the classification model. For example, He and Wu (2019) proposed a weighted logistic regression transfer learning algorithm based on Euclidean Space (EA), which aligns the EEG data of the source domain and target domain in the data preprocessing stage to reduce the differences between signals. In the feature extraction stage, the CSP algorithm is used to extract the feature values of different subjects, and then the Kullback-Leibler (KL) divergence of these feature values is calculated. Finally, the KL is used to adjust the Linear Discriminant Analysis (LDA) of the transfer learning for classification, achieving an average classification accuracy of 73.5%. However, this EA-CSP-LDA algorithm is not sensitive enough to non-linear data, resulting in poor classification performance. In addition, Zhou and Tian (2021) proposed a transfer multi-layers convolutional neural networks (TMCNN) algorithm, which first constructs a transfer network model capable of learning a large number of common features of the source domain, and then connects convolutional-pooling blocks to form four convolutional network structures with different depths. These structures are parallelly fused as a multi-level fusion feature extractor, and finally classified to achieve an average classification accuracy of 80.9%. However, this algorithm requires a large amount of data for model training, and the model is prone to overfitting when the dataset is small.

Due to the limited generalization ability of a single classifier, it is easy to overfit, so some researchers proposed to use ensemble learning to construct classifier. For example, Li et al. (2011) used a BP neural network as a weak classifier under the AdaBoost ensemble learning framework to form the BP-AdaBoost basic network classifier model. The model first extracts EEG features based on the Hilbert-Huang transform, and then introduces a forgetting factor to improve the AdaBoost algorithm, enhancing its temporal correlation by changing the initial weights of the samples. Finally, the BP neural network is used as a weak classifier and integrated into the BP-AdaBoost classifier. This model improves the classification accuracy by 23.42% compared to the traditional BP neural network, and achieves an average classification accuracy of 81.1%. However, the training time of BP neural network is long and it is prone to getting stuck in local optimal solutions, leading to a decrease in the performance of the classifier.

Inspired by the above studies, in order to break through the limitations of single domain EEG features to varying degrees, provide more comprehensive and detailed EEG analysis, and to simultaneously reduce the impact of small sample sizes of MI-EEG samples from individual subjects and inter-individual differences across subjects on MI task classification models, this paper proposes an EEG joint feature classification algorithm based on instance transfer and ensemble learning. The algorithm includes three stages: the first stage is preprocessing of source and target domain data; the second stage is to use the CSP algorithm and Power Spectral Density (PSD) to extract spatial and frequency domain features, respectively, and to combine the two features as EEG joint features, so as to fully explore EEG and enrich EEG features, and improve MI identification performance (Bin et al., 2019; Qiu et al., 2022); The third stage is to classify MI-EEG using an ensemble learning algorithm based on kernel mean matching (KMM) (Xing et al., 2007) and adaptive enhancement of transfer learning (TrAdaBoost) (Dai et al., 2007). Firstly, the KMM algorithm is used to adjust the sample weights to make the feature distribution of the source domain closer to that of the target domain. Then, the obtained weight sample matrix is used as the initialization weight matrix of the TrAdaBoost algorithm to initialize the training samples. Next, several weak classifiers are trained by using the initialized training sample data, and finally a strong classifier is integrated by weighted voting strategy. The algorithm makes full use of the EEG joint features and eliminates the individual differences of different subjects as much as possible. Repeated validation and testing show that the proposed algorithm can improve the classification performance of MI-EEG.

## 2. Materials and preprocessing

### 2.1. Data description

The MI-EEG data used in this paper is obtained from BCI Competition IV Dataset 2a and BCI Competition IV Dataset 2b (Tangermann et al., 2012).

The BCI Competition IV Dataset 2a is a dataset that collected EEG signals from 22 electrodes and recorded the locations of three electrooculogram (EOG) scalp electrodes for nine subjects. During the experiment, the subjects were asked to perform four different motor imagery tasks involving the left hand, right hand, foot, and tongue. Subjects sat in a comfortable chair and completed sessions consisting of six runs, with 48 motor imagery trials per run. In total, each session had 288 trials, with 72 trials performed for each type of motor imagery task.

The BCI Competition IV Dataset 2b is a dataset that collected EEG signals from three electrodes (C3, Cz, and C4) and recorded the locations of three EOG scalp electrodes for nine subjects. During the experiment, the subjects were asked to perform two different motor imagery tasks involving the left hand and right hand. Subjects sat in a comfortable chair and completed sessions consisting of six runs, with 20 motor imagery trials per run. In total, each session had 120 trials, with 60 trials performed for each type of motor imagery task.

**FIGURE 1:**
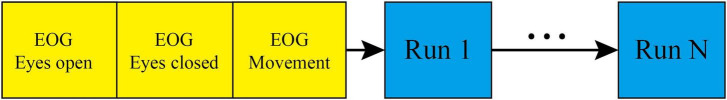
Timing scheme of one session.

**FIGURE 2:**
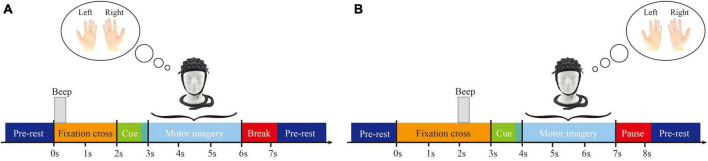
Timing scheme of the experimental paradigm. **(A)** Dataset 2a. **(B)** Dataset 2b.

This study only used data from motor imagery tasks involving the left and right hands. Prior to the start of each session, a 5 min EOG recording was performed to eliminate the influence of eye movement artifacts, followed by the start of the run. During each trial, subjects were required to fixate on the screen for 2 s before engaging in 4 s of motor imagery. The timing scheme of one session is shown in Figure 1, and the timing scheme of the experimental paradigm is shown in Figure 2. In addition, a sampling frequency of 250 Hz and a bandpass and a bandpass filter of 0.5–100 Hz were used during the experiment, along with a sensitive amplifier of 100 μV and a 50 Hz notch filter.

**FIGURE 3:**
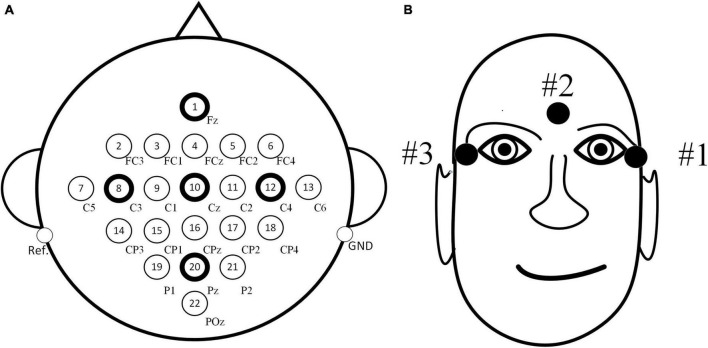
Distribution maps of electrode positions. **(A)** Positions of electroencephalography (EEG) electrodes. **(B)** Positions of electrooculogram (EOG) electrodes.

In the experiment, a total of 22 scalp electrodes based on the international 10–20 system were used, including Fz, FC3, FC1, FCz, FC2, FC4, C5, C3, C1, Cz, C2, C4, C6, CP3, CP1, CPz, CP2, CP4, P1, Pz, P2, and POz, left mastoid reference, right mastoid grounding, as shown in Figure 3A. In addition, three EOG electrodes were used, as shown in Figure 3B.

### 2.2. Data preprocessing

**FIGURE 4:**
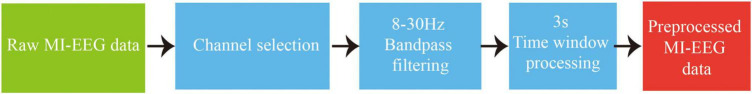
Data preprocessing workflow.

In the data preprocessing process of this study, the main steps used include channel selection, bandpass filtering, and time window processing. The detailed operation is as follows: In the channel selection step, the data recorded from the three EOG channels need to be removed, and the remaining EEG channels are used. As the relevant rhythmic signals during motor imagery are divided into μ rhythm signal of 8–12 Hz and β rhythm signal of 13–30 Hz (Chen et al., 2022), in the bandpass filtering step, a bandpass filter of 8–30 Hz is used to filter the selected EEG to improve the signal-to-noise ratio. Based on the experimental paradigm, each trial includes 4 s of motor imagery task time. Additionally, in the time window processing step, EEG corresponding to the motor imagery task time of 0.5–3.5 s after the cue are extracted, so as to better adapt to the needs of subsequent analysis by using the corresponding data from the 3 s time window in each trial, and improve the efficiency of data processing. The data preprocessing workflow is illustrated in Figure 4.

## 3. Related work

### 3.1. Spatial-frequency joint feature extraction

In order to fully explore MI-EEG signals and enrich EEG features, it is necessary to use multiple feature extraction methods to extract various types of features. This study used the CSP algorithm and PSD algorithm to extract spatial domain features and frequency domain features, respectively, and combined the two features as EEG joint features to provide more detailed and accurate EEG features, which could offer more comprehensive data support for subsequent signal processing and analysis.

#### 3.1.1. Spatial domain feature extraction based on CSP

**FIGURE 5:**
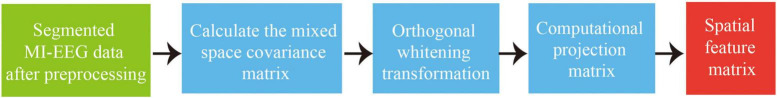
Common Spatial Pattern (CSP) feature extraction process.

common space mode is an algorithm for spatial filtering feature extraction that is extensively applied to binary classification MI-EEG tasks. It takes advantage of the characteristic of asymmetric energy differences in spatial brain areas during motor imagery, and projects the two types of EEG onto a subspace to decompose them into different spatial patterns. The fundamental principle of CSP algorithm involves diagonalization of matrices to obtain an optimal set of spatial filters, and then project the feature matrix to maximize the difference in variance between the two types of signals. This ultimately extracts spatial feature vectors with high discrimination ability, making it suitable for distinguishing between two types of MI tasks. The main process is shown in Figure 5.

(1) Sorting and segmenting the preprocessed data._*F_N_*=[*f*_1_(*t*),*f*_2_(*t*),…,*f_k_*(*t*),]^*T*^_ represents the signal of experiment _*N*_, with _*k*_ electrodes and _*n*_ trials per experiment. _*Trace*(.)_ is used to solve the matrix rank. The mixed-space covariance matrix _*H_c_*_ is calculated as follows:


(1) $$H_{c}=\frac{1}{n}\,\left(\sum \frac{\left(F_{1}\,F_{1}^{T}\right)}{\text{trace}\,\left(F_{1}\,F_{1}^{T}\right)}+\sum \frac{\left(F_{2}\,F_{2}^{T}\right)}{\text{trace}\,\left(F_{2}\,F_{2}^{T}\right)}\right)$$

(2) The mixed covariance space matrix _*H_c_*_ is subjected to orthogonal whitening transformation, where *I*. represents the identity matrix, to obtain the whitened matrix _*R*_ that satisfies the following condition.


(2) $$R\,H_{c}\,R^{T}=I$$

(3) Performing Singular Value Decomposition (SVD) on eigenvalue _*C_1_, C_2_*_ with _*C_1_=RH_1_R^T^ , C_2_=RH_2_R^T^*_ ,yields the diagonal matrix _*P*_ and the orthogonal matrix _*Q*_:


(3) $$C_{j}=QP_{j}Q^{T}\;\left(j=1,2\right)$$

(4) As inferred from (3), _*I*=*C*_1_+*C*_2_, *P*_2_=*I*–*P*_1__ . When any element in _*C_j_*_ approaches _*I*_, the other elements of _*C_j_*_ will approach the zero matrix. This maximizes the variance differences between the two classes of signals. Therefore, the spatial filtering _*Z*_ of the N-th EEG is:


(4) $$Z=Q^{T}\,Q\,F_{N}$$

(5) Spatial feature _*f_j_*_ of MI-EEG:


(5) $$f_{j}=\frac{v\,a\,r\,\left(Z_{j}\right)}{\sum_{j=1}^{m}v\,a\,r\,\left(Z_{j}\right)}$$

Among them, _*Z_j_*_ is the projection of _*F_N_*_ onto the spatial filter, _*m*_ is the number of selected feature parameters, _*f*_ is the feature value. _*Var*(.)_ is the variance of the matrix.

#### 3.1.2. Frequency domain feature extraction based on PSD

**FIGURE 6:**
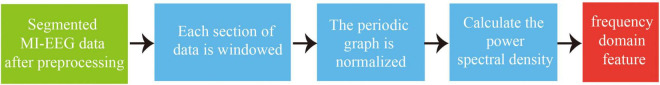
Power spectral density (PSD) feature extraction process.

As the EEG are non-stationary random signals, this study used the Welch method (Bin et al., 2019) in the PSD algorithm to extract frequency domain features from the EEG. This method reduces the random fluctuations in signals to obtain results with smaller variance and smoother curves, aiming to extract high-discriminative frequency domain features. The algorithm involves segmenting complex signals, applying window functions to calculate power spectral density, and finally outputting the averaged results. The main process is illustrated in Figure 6.

(1) Segment the EEG _*f(m), m=0, 1, …, K–1*_ into _*R*_ segments, each containing _*N*_data points, where the length of the EEG is _*K*_. Segment _*j*_ of data can be represented as:


(6) $$f_{j}\,\left(m\right)=f\,\left(j\,N-N+n\right),0\leq j\leq R,0\leq m\leq N$$

(2) Apply a windowing function _*w(m)*_ to each segment of data, and then normalize the resulting periodogram _*P(λ)*_.


(7) $$P\,\left(\lambda \right)=\frac{1}{N\,U}\,{\left|\sum_{m=0}^{N-1}f_{j}\,\left(m\right)\,w\,\left(m\right)\,e^{-i\,2\,\pi \,\lambda }\right|}^{2},j=1,2,\ldots ,N-1$$

(3) The final estimate of power spectral density _*P_welch_(λ)*_ is:


(8) $$P_{w\,e\,l\,c\,h}\,\left(\lambda \right)=\frac{1}{R}\,\sum_{j=1}^{R}P\,\left(\lambda \right)$$

Where _*U*_is the normalization factor, _λ_ is the frequency, _*P*(λ)_ is the normalized periodogram, and _*P_welch_*(λ)_ is the power spectral density.

### 3.2. Ensemble learning classification

In this study, an ensemble learning algorithm based on KMM and TrAdaBoost (K-T) was used to classify MI-EEG. First, the sample weight matrix obtained by the KMM algorithm was used as the initial sample weight matrix for the TrAdaBoost algorithm. Then, several weak classifiers were trained using the initialized training samples. Finally, a strong classifier was obtained using a weighted voting strategy.

#### 3.2.1. KMM algorithm

Due to individual differences, different subjects may have different feature distributions in their EEG. KMM can adjust the sample weights to make the feature distribution of the source domain similar to that of the target domain. Firstly, the raw feature Space was mapped to Reproducing Kernel Hilbert Space (RKHS) (Gertton et al., 2012), and then the mean difference between the source domain samples and the target domain samples in the RKHS was computed, resulting in a set of weight parameter matrices. These matrices can be used to re-assign weights to the training samples, aiming to achieve the feature distribution of the source domain approaching that of the target domain under the action of the nuclear space. The process can be expressed as follows:


(9) $$\min{||\frac{1}{n}\,\sum_{i=1}^{n}\beta_{i}\,\Phi \,\left(x_{i}^{s}\right)-\frac{1}{m}\,\sum_{i=1}^{m}\Phi \,\left(x_{i}^{t}\right)||}_{H}^{2}$$

Where,$x_{i}^{s}$ is a set of samples from the source domain, _*i=1, 2, … , n*_, $x_{i}^{t}$ is a set of samples from the target domain, _*i=1, 2, … , m*_. _β_i_∈[0,1]_ is the weight for the _*i–th*_ sample from the source domain. _*H*_ is a RKHS with a feature kernel _*K*_, _Φ(∙)_ is the mapping function from the original space to the RKHS, and satisfies the following relation: _(Φ(*x*),Φ(*y*))_*H*_=*K*(*x,y*),_ where _*K(x,y)*_ is the Gaussian kernel function.


(10) $$K\,\left(x,y\right)=\exp\left(\frac{-{||\text{x-y}||}^{2}}{2\,\sigma^{2}}\right)$$

Where, _σ_ represents the size of the Gaussian kernel. By combining equations (9) and (10), we can finally obtain the mean difference between each source domain and target domain:


(11) $$\min\left(\frac{1}{n^{2}}\,{\left(\sum_{i=1}^{n}\beta_{i}\,\Phi \,\left(x_{i}^{s}\right)\right)}^{2}-\frac{2}{n\,m}\,\sum_{i=1}^{m}\beta_{i}\,\Phi \,\left(x_{i}^{s}\right)\,\sum_{i=1}^{m}\Phi \,\left(x_{i}^{t}\right)+c\right)$$

Where, _σ_ represents a constant.

#### 3.2.2. TrAdaBoost algorithm

Instance transfer learning selects samples from the source domain that are consistent with the target sample space distribution, and improves the performance of the model on the target task by transferring information from the source domain. TrAdaBoost algorithm is an instance transfer learning algorithm based on AdaBoost algorithm. During its operation, it uses source domain samples and some target domain samples as training set samples, and the remaining target domain samples as test set. The model is trained based on the training samples, and then tested on the test set. Meanwhile, Hedge algorithm weighting mechanism is used to deal with samples from the auxiliary domain (Chen et al., 2021). That is, in each iteration of training, if the model misclassifies a sample from the source domain, we consider that this sample have a large difference with the target domain sample, and therefore need to reduce the weight of this sample. By multiplying this sample with a weight between 0 and 1, the impact of this sample on the classification model will be reduced in the next iteration through the weight value. After a series of iterations, the weight of the source domain samples that are helpful for classifying the target domain will be increased, while the weights of other source domain samples will be decreased. This achieves the goal of enhancing valuable auxiliary samples and gradually weakening the distribution-dissimilar auxiliary samples. Finally, by further training several weak classifiers based on the modified weight data and weighting them, a strong classifier is obtained, thereby improving the classification performance of the model on the target task by transferring information from the source domain.

Let $A_{s}=\left\{\left(x_{i},y_{i}\right)\right\}$ be the sample from the source domain with i=1, 2, ... , n, and $A_{t}=\left\{\left(x_{i},y_{i}\right)\right\}$ be the sample from the target domain with i=n+1, n+2, ... , n+m, where _*y*_i__ represents the sample label and _*x*_i__ represents the sample instance. Let _*T*_ be the testing data. The sample space of _*T*_ and _*A_s_*_ are of the same distribution, while _*T*_ and _*A_t_*_ are of different distributions. During operation, a classifier will be trained with a large number of _*A_s_*_ and a small number of _*A_t_*_ to minimize classification errors on _*T*_.

(1) Initialize the weights of training samples_*A={A_s_,A_t_}*_:


(12) $$w_{i}^{1}=\left\{ \begin{array}{c} 1/n\,i=1,2,3\,\ldots ,n\\ 1/m\,i=n+1,\ldots ,n+m \end{array}\right.$$

(2) Setting the parameters _β_ for the auxiliary samples:


(13) $$\beta =1/\left(1+\sqrt{2\,\ln n/N}\right)$$

(3) Utilize *A*, *T*, and normalized weight *w* to train a weak classifier _*h_t_*_.

(4) Calculate the error rate _ε_t__ of _*h_t_*_ using the training data set in the target domain _*A_t_*_ :


(14) $$\varepsilon_{t}=\sum_{i=n+1}^{n+m}\frac{w_{i}|h_{t}\left(x_{i}\right)-c\left(x_{i}\right))|}{\sum w_{i}^{t}}$$

(5) Setting _β_t_ =ε_t_ /(1–ε_t_)_ results in a new weight value _*w*_ of:


(15) $$w_{i}^{i+1}=\left\{ \begin{array}{c} w_{i}^{t}\,\beta^{\left|h_{t}\,\left(x_{i}-c\,\left(x_{i}\right)\right)\right|}\;i=1,2,3,\ldots ,n\\ w_{i}^{t}\,\beta_{t}^{-\left|h_{t}\,\left(x_{i}-c\,\left(x_{i}\right)\right)\right|}\,i=n+1,\ldots ,n+m \end{array}\right.$$

(6) After _*N*_ iterations, the resulting strong classifier _*h(x)*_ is:


(16) $$h\,\left(x\right)=\left\{ \begin{array}{c} 1,\sum_{t=\left\lceil N/2\right\rceil }^{N}\ln\left(1/\beta_{t}\right)\,h_{t}\,\left(x\right)\geq \frac{1}{2}\,\sum_{t=\left\lceil N/2\right\rceil }^{N}\ln\left(1/\beta_{t}\right)\\ 0,o\,t\,h\,e\,r \end{array}\right.$$

### 3.3. Our algorithm

**FIGURE 7:**
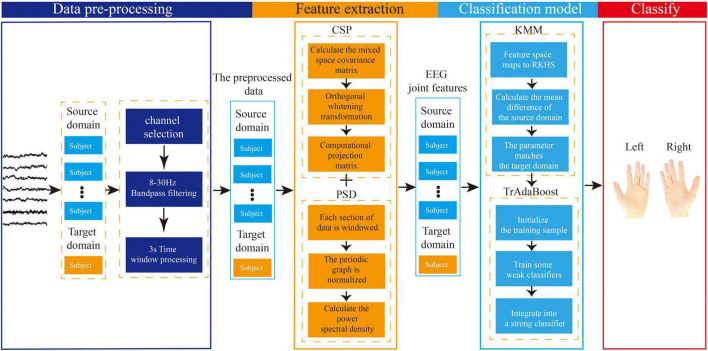
Flowchart of electroencephalography (EEG) joint feature classification algorithm based on instance transfer and ensemble learning.

The proposed EEG joint feature classification algorithm based on instance transfer and ensemble learning in this study consists of three parts: data preprocessing, spatial-frequency joint feature extraction, and ensemble learning classification. The main process is as follows, as shown in Figure 7.

(1) In the data preprocessing part, channel selection, band-pass filtering, and time window processing will be performed on the raw EEG data from the source and target domains.

(2) In the feature extraction part, the preprocessed data from the source and target domains will be used for feature extraction. The spatial features will be extracted based on the CSP algorithm, and the frequency features will be extracted based on the PSD algorithm using Welch’s method. Finally, the spatial and frequency features will be combined to form the joint EEG features.

(3) In the ensemble learning classification part, the sample weights of the joint features obtained from the source and target domains will be adjusted using the KMM algorithm to make the source and target domains as close as possible, and the sample weight matrix will be obtained. Then, this matrix will be used as the initialization weight matrix in the TrAdaBoost algorithm and used to initialize the training samples. Next, multiple weak classifiers will be trained using the initialized training data, and finally, a strong classifier will be obtained using a weighted voting strategy based on the weights of each weak classifier, achieving the goal of using ensemble learning for MI-EEG classification.

## 4. Experiment analysis

### 4.1. Result evaluation index

In this study, The Accuracy (ACC) was selected as an indicator to evaluate the performance of the classification model, and the calculation formula is as follows:


(17) $$A\,c\,c=\frac{T\,P+T\,N}{T\,P+T\,N+F\,P+F\,N}$$

Where, _*TP*_ and _*TN*_ represent the number of samples correctly classified as positive labels and negative labels, respectively. _*FP*_ and _*FN*_ are the number of samples misclassified as positive labels and negative labels, respectively.

### 4.2. Comparative experiment and results analysis

In this study, EEG data from nine subjects (A01, A02,…, A09) in the BCI Competition IV Dataset 2a, as well as EEG data from nine subjects (B01, B02,…, B09) in the BCI Competition IV Dataset 2b, will be selected as research data. During each experiment targeting each dataset, EEG data from eight subjects were selected as source domain data, and data from another subject was selected as target domain data. To validate the effectiveness of our proposed EEG joint feature classification algorithm based on instance transfer and ensemble learning (Ours), several comparative experiments will be designed, including CSP+SVM, PSD+SVM, CSP+PSD+SVM, CSP+PSD+KMM, and CSP+PSD+TrAdaBoost.

(1) CSP+SVM, PSD+SVM, and CSP+PSD+SVM were taken as a group of comparative experiments. The spatial domain features, frequency domain features, and EEG joint features were, respectively classified by SVM algorithm. The average classification accuracies for these three experiments are 66.8, 58.9, and 73.2% in Dataset 2a, and 64.7, 60.4, and 70.1% in Dataset 2b, respectively. The CSP+PSD+SVM achieved the best classification effect, indicating that the joint spatial and frequency domain features can improve the classification performance of MI-EEG. However, its classification accuracy is lower than Ours by about 18 and 13% in Dataset 2a and Dataset 2b, respectively, mainly due to the large individual differences in MI-EEG among different subjects, and the trained models of other subjects cannot be well-applied to the current subject. This also confirms that the K-T algorithm utilizing instance transfer method can effectively solve the problem of individual differences among different subjects.

(2) CSP+PSD+TrAdaBoost algorithm: The average classification accuracy obtained by using the TrAdaBoost algorithm to classify the joint features of EEG is only 81.3 and 76.2% in Dataset 2a and Dataset 2b, respectively, which is lower than the classification accuracy of Ours. The main reason is that the distribution of data in source domain and target domain is different, and the TrAdaBoost algorithm uses the same weight as the initial weight matrix during classification initialization, which leads to unreasonable sample weights. In this case, if KMM algorithm is used to adjust the sample weight matrix, the data can be distributed more reasonably.

(3) CSP+PSD+KMM algorithm: The average classification accuracy obtained by using the KMM algorithm to classify the joint features of EEG is only 84.3 and 76.4% in Dataset 2a and Dataset 2b, respectively, which is lower than the classification accuracy of Ours. The main reason is that KMM needs to estimate a kernel density ratio, and the error of this ratio estimate negatively affect the performance of domain adaptation. In this case, if the TrAdaBoost algorithm is used to reassign different weights to the source domain and target domain to reduce the influence of estimation errors in KMM algorithm, the performance of domain adaptation can be improved.

**FIGURE 8:**
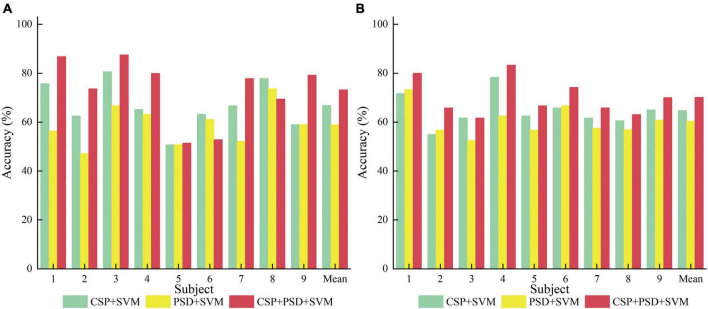
Comparison of classification accuracy among three methods for nine subjects in the first group of experiments. **(A)** Dataset 2a. **(B)** Dataset 2b.

From Table 1, Figure 8, it can be seen that for the same classification method, using the EEG joint features as the feature vector can significantly improve the average classification accuracy by 6–14% and 5–10% in Dataset 2a and Dataset 2b, respectively, compared to using the feature vector from a single domain. It shows that EEG joint features can break through the limitations of single domain EEG features, provide more comprehensive and detailed EEG analysis, and improve the classification performance of MI-EEG.

**TABLE 1:** Comparison of classification accuracy among the three methods for nine subjects in Dataset 2a and Dataset 2b in the first group of experiments.

Subject	Method	CSP+SVM	PSD+SVM	CSP+PSD+SVM
Dataset 2a	A01	75.7%	56.3%	86.8%
	A02	62.5%	47.2%	73.6%
	A03	80.6%	66.7%	87.5%
	A04	65.2%	63.2%	79.9%
	A05	50.7%	50.7%	51.4%
	A06	63.2%	61.1%	52.8%
	A07	66.7%	52.1%	77.8%
	A08	77.8%	73.6%	69.4%
	A09	59.0%	59.0%	79.2%
	Mean ± SD	(66.8 ± 9.1)%	(58.9 ± 7.9)%	(73.2 ± 12.5)%
Dataset 2b	B01	71.7%	73.3%	80.0%
	B02	55.0%	56.7%	65.8%
	B03	61.7%	52.5%	61.7%
	B04	78.3%	62.5%	83.3%
	B05	62.5%	56.7%	66.7%
	B06	65.8%	66.7%	74.2%
	B07	61.7%	57.5%	65.8%
	B08	60.6%	56.9%	63.1%
	B09	65.0%	60.8%	70.0%
	Mean ± SD	(64.7 ± 6.4)%	(60.4 ± 5.8)%	(70.1 ± 7.1)%

**FIGURE 9:**
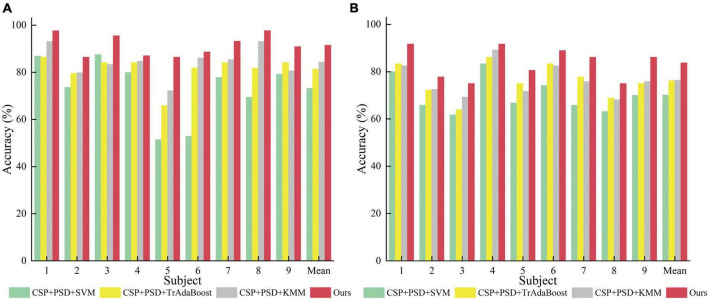
Comparison of classification accuracy between the proposed method and the comparative experimental method. **(A)** Dataset 2a. **(B)** Dataset 2b.

According to Tables 2, 3, and Figure 9, when EEG joint features are used as feature vectors, the average classification accuracy obtained by Ours algorithm in Dataset 2a is 18.3, 10.2, and 7.2% higher than that obtained by SVM algorithm, TrAdaBoost algorithm and KMM algorithm, respectively. They were 13.6, 7.5, and 7.3% higher in Dataset 2b. It is explained that Ours, considering that TrAdaBoost algorithm can improve the performance of classifier and reduce the classification error rate through sample weighting. At the same time, it is also considered that the KMM algorithm can further improve the matching of sample distribution by distributing the importance weight of samples. Therefore, by using K-T ensemble learning, several weak classifiers can be integrated into strong classifiers through sample importance weight allocation and weighted classifiers, so as to eliminate individual differences among different subjects, so as to improve the generalization ability of classification model and improve the classification performance of MI-EEG.

**TABLE 2:** Comparison of classification accuracy between the proposed method and the comparative experimental method in Dataset 2a and Dataset 2b.

Subject	Method	CSP+PSD+SVM	CSP+PSD+TrAdaBoost	CSP+PSD+KMM	Ours
Dataset 2a	A01	86.8%	86.4%	93.1%	97.6%
	A02	73.6%	79.5%	79.8%	86.4%
	A03	87.5%	84.1%	83.3%	95.5%
	A04	79.9%	84.1%	84.7%	87.0%
	A05	51.4%	65.9%	72.2%	86.4%
	A06	52.8%	81.8%	86.1%	88.6%
	A07	77.8%	84.1%	85.4%	93.2%
	A08	69.4%	81.8%	93.1%	97.7%
	A09	79.2%	84.1%	80.6%	90.9%
	Mean ± SD	(73.2 ± 12.5)%	(81.3 ± 5.8)%	(84.3 ± 5.8)%	(91.5 ± 4.4)%
Dataset 2b	B01	80.0%	83.3	82.5	91.7
	B02	65.8%	72.2	72.5	77.8
	B03	61.7%	63.9	69.2	75.0
	B04	83.3%	86.1	89.2	91.7
	B05	66.7%	75.0	71.7	80.6
	B06	74.2%	83.3	82.5	88.9
	B07	65.8%	77.8	75.8	86.1
	B08	63.1%	68.8	68.1	75.0
	B09	70.0%	75.0	75.8	83.3
	Mean ± SD	(70.1 ± 7.1)%	(76.2 ± 6.8)%	(76.4 ± 6.6)%	(83.7 ± 6.3)%

**TABLE 3:** T-test results of the proposed method and the comparative experimental method.

Paired T-test	*P*-value	
	Dataset 2a	Dataset 2b
Ours vs. CSP+PSD+SVM	0.001	0.000
Ours vs. CSP+PSD+TrAdaBoost	0.000	0.000
Subject vs. CSP+PSD+KMM	0.001	0.000

*P* < 0.05 indicates significant results.

### 4.3. Comparison with state-of-the-art methods

**FIGURE 10:**
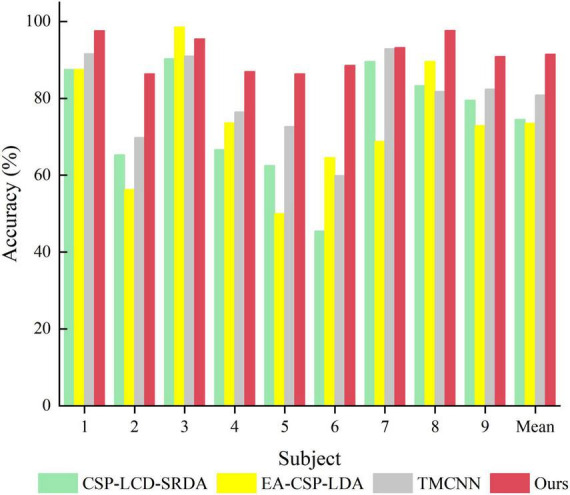
Comparison of classification accuracy between the proposed method and other methods.

In order to verify the superiority of the algorithm proposed in this paper, it is compared with algorithms proposed by other researchers in the same data set (BCI Competition IV Dataset 2a), and the experimental results are shown in Table 4 and Figure 10. As can be seen from the table, the accuracy of subjects five and six is very low in other literatures, but in Ours, it is significantly improved by more than 13%. In addition, compared with the methods of other researchers, the average classification accuracy of Ours was increased by more than 10%, indicating that Ours outperforms other algorithms in the classification performance of MI-EEG.

**TABLE 4:** Comparison of classification accuracy of the method presented in this paper and the methods proposed by other researchers on brain-computer interface (BCI) competition IV dataset 2a.

Subject	Method	CSP-LCD-SRDA	EA-CSP-LDA	TMCNN	Ours
Dataset 2a	A01	87.5%	87.5%	91.6%	97.6%
	A02	65.3%	56.3%	69.8%	86.4%
	A03	90.3%	98.6%	91.0%	95.5%
	A04	66.7%	73.6%	76.5%	87.0%
	A05	62.5%	50.0%	72.7%	86.4%
	A06	45.5%	64.6%	59.9%	88.6%
	A07	89.6%	68.8%	92.9%	93.2%
	A08	83.3%	89.6%	81.8%	97.7%
	A09	79.5%	72.9%	82.4%	90.9%
	Mean ± SD	(74.5 ± 14.5)%	(73.5 ± 15.0)%	(80.9 ± 10.5)%	(91.5 ± 4.4)%

## 5. Conclusion

During the process of motor imagery, there are asymmetric energy differences in the spatial brain area. Therefore, researchers usually use the CSP algorithm to extract distinctive spatial features from multi-channel EEG. In addition, since EEG are mainly rhythmic signals, ERD and ERS occur within specific frequency bands, and CSP algorithm directly performs spatial filtering on frequency domain signals, so frequency domain information is crucial for MI task classification and cannot be ignored. Therefore, in order to fully explore EEG and enrich EEG features and improve the recognition of MI signals, this paper proposes to use CSP and PSD to extract spatial and frequency domain features, respectively, and combine them to obtain EEG joint features, providing a more comprehensive and detailed analysis of EEG. In addition, the sample size of individual training for MI-EEG is small, and there are great individual differences among different subjects. This paper proposes a joint feature classification algorithm for EEG based on instance transfer and ensemble learning. After obtaining the EEG joint features, KMM and TrAdaboost are combined, and an ensemble learning strategy is used to integrate a classifier with strong generalization ability. The experimental results show that the algorithm has an average accuracy of 91.5 and 83.7% on Dataset 2a and Dataset 2b, respectively, which is significantly better than other algorithms and provides a new approach to solving the above problems. However, it may not be applicable to other EEG data such as P300 and emotion recognition data. Therefore, in future research, we can make the algorithm applicable to other EEG signal data by improving the alignment between the source domain and the target domain.

## Data availability statement

The original contributions presented in this study are included in the article/supplementary material, further inquiries can be directed to the corresponding authors.

## Author contributions

XW and XD designed the research. XW wrote the manuscript. RH and ML collected the data and supervised the study. XC, YL, and QH supervised and revised the manuscript. All authors contributed to the article and approved the submitted version.
